# Expression of proinflammatory cytokines and proinsulin by bone marrow-derived cells for fracture healing in long-term diabetic mice

**DOI:** 10.1186/s12891-023-06710-5

**Published:** 2023-07-18

**Authors:** Hitomi Fujikawa, Hideto Kojima, Tomoya Terashima, Miwako Katagi, Takafumi Yayama, Kosuke Kumagai, Kanji Mori, Hideki Saito, Shinji Imai

**Affiliations:** 1 Department of Orthopaedic Surgery, Shiga University of Medical Science, Setatsukinowa-cho, Otsu, 520-2192 Shiga Japan; 2 Department of Stem Cell Biology and Regenerative Medicine, Shiga University of Medical Science, Otsu, 520-2192 Shiga Japan

**Keywords:** Long-term hyperglycaemia, Fracture healing, TNF-α, Proinsulin, Bone marrow transplantation

## Abstract

**Background:**

Diabetes mellitus (DM) causes bone dysfunction due to poor bone quality, leading to severe deterioration in patient of quality of life. The mechanisms of bone metabolism in DM remain unclear, although chemical and/or mechanical factors are known to disrupt the homeostasis of osteoblasts and osteoclasts. The purpose of this study was to identify the changes of osteoblasts and osteoclasts under long-term hyperglycaemic conditions, using a mouse fracture model of long-term hyperglycemia (LT-HG).

**Methods:**

C57BL/6J mice and green fluorescent protein (GFP) -positive bone marrow transplanted C57BL/6J mice with LT-HG, maintained under a state of hyperglycaemia for 2 months, were used in this study. After the experimental fracture, we examined the immunohistochemical expression of proinsulin and tumor necrosis factor (TNF) -α at the fracture site. C57BL/6J fracture model mice without hyperglycaemia were used as controls.

**Results:**

In the LT-HG mice, chondrocyte resorption was delayed, and osteoblasts showed an irregular arrangement at the callus site. The osteoclasts were scattered with a decrement in the number of nuclei. The expression of proinsulin was confirmed in bone marrow derived cells (BMDCs) with neovascularization 2 and 3 weeks after fracture. Immunopositivity for TNF-α was also confirmed in immature chondrocytes and BMDCs with neovascularization at 2 weeks, and the number of positive cells was not decreased at 3 weeks. Examination of GFP-grafted hyperglycaemic mice showed that the majority of cells at the fracture site were GFP-positive. Immunohistochemistry showed that the rate of double positives was 15% for GFP and proinsulin and 47% for GFP and TNF-α.

**Conclusion:**

LT-HG induces an increase in the number of proinsulin and TNF-α positive cells derived from BMDCs. We suggest that proinsulin and TNF-α positive cells are involved in both bone formation and bone resorption after fracture under hyperglycaemic conditions, resulting in the delay of bone healing.

**Supplementary Information:**

The online version contains supplementary material available at 10.1186/s12891-023-06710-5.

## Background

The prevalence of diabetes mellitus (DM) is increasing globally due to lifestyle changes, urbanization, population growth, and aging demographics; the number of diabetic patients is expected to exceed 700 million by 2025 [1, 2]. However, the optimal treatment strategies for DM and DM-related complications, such as peripheral neuropathy, renal disorders, and increased risk of fractures, remain unclear. Bone strength consists of two factors; bone mass and bone quality, with poor bone quality considered more important than bone loss in diabetic bone dysfunction [3]. According to a prior meta-analysis by Vestergaard et al., patients with DM type 1 and 2 have an increased risk of hip fractures compared to non-diabetics [4]. In addition, the risk of delayed union or nonunion after fracture increases 2.2–3.4 fold [5, 6]. Therefore, diabetic bone dysfunction is one of the important factors that can decrease activities of daily living and quality of life.

The balance between bone formation and bone resorption is important for maintaining bone homeostasis. The causes of poor bone formation in DM may be due to increased bone marrow (BM) adiposity of mesenchymal stem cells (MSCs) and impaired differentiation and mineralization of osteoblasts [7–10]. In previous studies by Kojima et al. [11] and Terashima et al. [12], bone marrow cells (BMCs) were found to express proinsulin and tumor necrosis factor (TNF) -α, allowing them to fuse with each organ cell to cause cytopathy in diabetic hepatic, renal, and peripheral models of neuropathy. Furthermore, in a short-term hyperglycaemia mouse fracture model in which hyperglycaemia was maintained for 2 weeks, the sizes of the osteoclasts and the resorption pits formed were significantly smaller. Moreover, the expression of DC-STAMP, a putative pivotal gene for osteoclast fusion, decreased in osteoclasts in DM mice. Together, these results highlight the importance of impaired bone resorption due to osteoclast dysfunction [13]. DM patients are exposed to hyperglycaemia for long periods of time, ranging from years to decades. However, how proinsulin- and TNF-α-producing cells appear in the bone tissue of long-term diabetic patients and how they are able to alter the balance between bone formation and bone resorption remains to be elucidated.

In the present study, we induced fractures in long-term hyperglycaemia (LT-HG) mice, in maintained under a state of hyperglycemia for 2 months, and analyzed the histological changes in osteoblasts and osteoclasts that appeared in the callus, in addition to changes in the expression of proinsulin and TNF-α. In order to investigate the origin of these cells, bone marrow transplantation (BMT) of green fluorescent protein (GFP) -positive cells was performed, and their cell distribution was observed. The purpose of this research was to analyze the mechanisms of bone formation and bone resorption in a diabetic bone dysfunction model and to obtain new knowledge for the development of future treatment methods.

## Methods

### Mouse husbandry and grouping

This research was approved by the Institutional Review Boards of the authors’ affiliated institutions. Eight-week-old male C57BL/6J mice (wild type, Japan SLC, Inc., Shizuoka, Japan) and C57BL/6-Tg (UBC-GFP) 30Scha/J mice (GFP-Tg mice, Jackson Laboratory, Bar Harbor, ME, USA) mice were used for this study. Mice were housed in our university double-barrier facility under a 12-h light cycle, and allowed access to commercial chow and water *ad libitum*.

For investigation of osteoblasts, osteoclasts, and proinsulin and TNF-α-producing cells at the callus formation site, mice were randomly allocated to two groups: with (LT-HG positive; *n* = 10) or without (LT-HG negative; *n* = 10) exposure to LT-HG with streptozotocin (STZ). For the GFP-positive BMT models, mice were selected with LT-HG (GFP and LT-HG positive; *n* = 5) and without LT-HG (GFP positive and LT-HG negative; *n* = 5). Each mouse was subjected to histological and immuno-histochemical examination at 2 or 3 weeks after fracture, after euthanization by perfused fixation under deep anesthesia. (Supplemental Data. d)

### Long term hyperglycaemia model

Hyperglycaemia was induced using STZ (180 mg/kg body weight) (Nacalai Tesuque, Kyoto, Japan) injected into C57BL/6 J mice intraperitoneally. The control group mice without LT-HG were injected with citrate buffer (pH 4.5). Blood obtained from the tail vein was measured by Glutest-Ace (Sanwa kagaku, Nagoya, Japan). Hyperglycaemia was diagnosed when blood glucose levels (BGL) reached over 300 mg/dl. Maintenance of hyperglycaemia for up to 2 months was confirmed by glucose measurement every 2 weeks. (Supplemental Data. a). Mice with a blood glucose level of less than 300 mg/dl at any measurement were excluded from the study. Based on a study by Terashima et al., we defined 2 months of hyperglycaemia exposure as the LT-HG group because mice were shown to exhibit diabetic neuropathy during 2 months of hyperglycaemia exposure [12].

### Fracture model

**Fig. 1:**
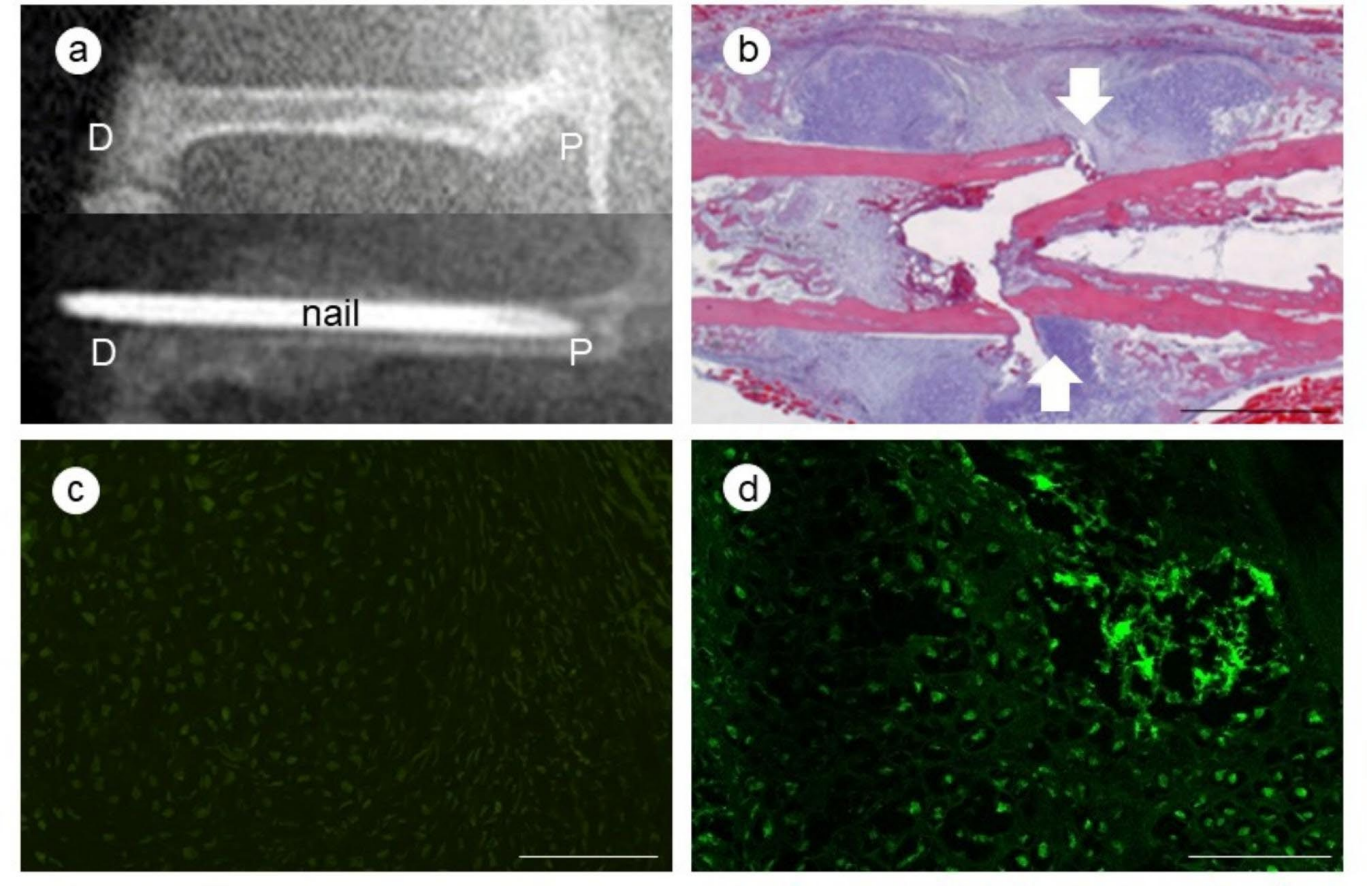
Overview of the creation of the bone fracture model. Bone radiological image of the femur: upper column, before insertion of intramedullary nail into femur: lower column, Insertion of a 23-gauge needle as an intramedullary nail for internal fixation after a fracture procedure. (P: proximal of femur, D: distal of femur) (**a**); histological extermination of the femur at fracture site. (White arrow: fracture level) (**b**) (bar: 1 mm); femur fracture site fluorescence microscope images after green fluorescent protein (GFP) bone marrow transplantation: GFP negative image (**c**), GFP positive image (**d**). (bar: 100 μm)

Mice were anesthetized by intraperitoneal administration of a mixed aesthetic (medetomidine 0.3 mg/kg, midazolam 4.0 mg/kg, butorphanol 5.0 mg/kg), then positioned laterally to make a transverse, open fracture of the right femur. A skin incision was made on the lateral side of the right femur, the muscle was incised in the direction of the muscle fibers, the femur was exposed, and the central femur was cut with scissors without periosteal dissection. A 23-gauge needle was inserted into the femur for internal fixation, and the wound was sutured (Fig. 1a, b). The mice were permitted full weight bearing activity immediately.

### Bone marrow transplantation model

Recipient C57BL/6J mice were irradiated (9 G× for 5 min) using an X-ray irradiation device (MBR-1520R, Hitachi Medical Corporation, Tokyo, Japan). The GFP-Tg mouse donors were euthanized by cervical dislocation, then the humerus, femur, and tibia were collected, and BMCs were washed out. The BMC suspension was centrifuged at 1500 rpm for 5 min, and the suspension was diluted with 0.1 M phosphate buffer saline (PBS) HBSS. Whole BMCs (4 × 10^6^ cells) were injected through the tail artery of a recipient mouse to make a GFP-positive BMT model (Fig. 1c, d).

### Histological and immunohistochemical examination

Mice were deeply anesthetized with the same aesthetic mixture described above, and perfused with PBS followed by a fixative containing 4% paraformaldehyde in 0.1 M phosphate buffer. After perfusion fixation, the intramedullary needle was removed, and the specimens were decalcified in ethylenediamine tetra acetic acid disodium for 1 week at 4 °C. The specimens were then dehydrated through a graded ethanol series, embedded in paraffin, and the sections were cut at a thickness of 4 μm. Hematoxylin-eosin (HE) staining of the callus was to performed to evaluate its morphology. Immunostaining was performed using the ABC method, with anti-OC antibody (1:100; ab93876, Abcam, Cambridge, UK), anti-RANK antibody (1:50; 64C1385, Abcam, Cambridge, UK), anti-proinsulin antibody (1:1000; Progen Biotechnik, Heidelberg, Germany), and anti-TNF-α antibody (1:100; ab6671, Abcam, Cambridge, UK). The specimens were examined under an optical microscope (FXA, Nikon, Tokyo, Japan), and sections were evaluated and measured using Image-Pro Plus software (Media Cybernetics, Rockville, MD, USA).

Frozen sections of non-decalcified tissue were prepared using the Kawamoto film method [14]. Mice were anesthetized and perfusion-fixed using the same method as described above. The collected right femur was placed in an embedding agent (SCEM, Section-Lab Co. Ltd., Hiroshima, Japan), submerged in hexane, and cooled with liquid nitrogen to create a freeze-embedded block. The blocks were trimmed in a cryostat using a tungsten carbide knife (TC-65, Leica Microsystems Co., Ltd., Hiroshima, Japan), and a film (Cryofilm Type 2 C, Section-Lab Co., Ltd., Hiroshima, Japan) was applied to the thin section surface to prepare sections of 4 μm thickness. The cryosections were dried at room temperature for 60 s, and the sections were placed in 100% alcohol. Staining was performed using the floating method, with the following antibodies: anti-proinsulin antibody (1:1,000; Progen Biotechnik, Heidelberg, Germany) and anti-TNF-α antibody (1:100; ab6671, Abcam, Cambridge, UK). Alexa 405 anti-rabbit IgG antibody (ab175651, Abcam, Cambridge, UK) and Alexa 647 anti-guinea pig IgG antibody (ab150187, Abcam, Cambridge, UK) were used as fluorescence-labelled secondary antibodies. After staining, the sections were polymerized and cured with cover glass using encapsulating materials (SCMM-R2, Section-Lab Co., Ltd., Hiroshima, Japan) and encapsulation equipment (UV Quickcryosection Mounter, Section-Lab Co., Ltd., Hiroshima, Japan). Sections were evaluated with a confocal laser scanning microscope (TCS SP8 X, Leica, Wetzlar, Germany), and images were merged for cell count and semiquantitative evaluation.

### Statistical analysis

Correlation analyses of cell counts via immunohistochemical examination were performed using the Mann–Whitney U test. Differences were considered significant at *P* < 0.05. All analyses were performed using SPSS software version 25 (IBM Corp., Armonk, NY, USA).

## Results

At the beginning of the experiment, the average body weight (BW) of the mice was 21.66 g (± 0.843) for the non-hyperglycaemia control group (HT-LG^–^) and 20.98 g (± 0.72) for the long-term hyperglycaemia group (LT-HG^+^). After 2 months, the BW was 26.32 g (± 0.865) for the HT-LG^–^ group and 20.5 g (± 1.78) for the LT-HG^+^ group (Supplemental Data. b). The mean BGL of the HT-LG^–^ group remained at 120–140 mg/dl (± 17.92–23.42) during the experimental period, while that of the LT-HG^+^ group was 119.6 mg/dl (SD 13.45) before STZ injection and 360–390 mg/dl (± 50.95–66.16) afterward (Supplemental Data. a).

**Fig. 2:**
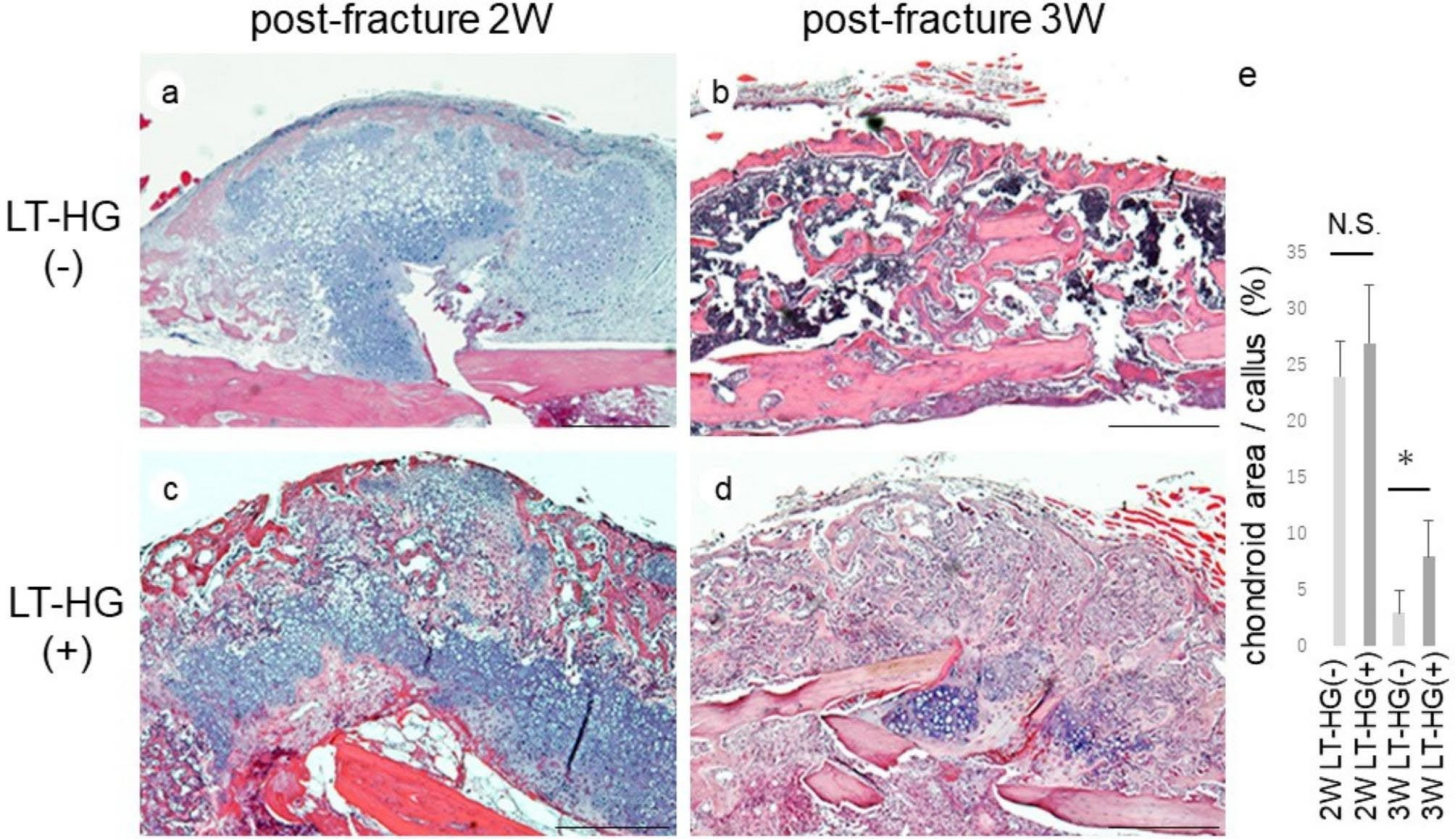
Callus at the femur fracture site. Hematoxylin-eosin (HE) images of callus formation without long-term hyperglycaemia (LT-HG) at 2 weeks (2 W) after fracture (**a**), 3 weeks (3 W) after the fracture (**b**), with LT-HG at 2 W after fracture (**c**), and at 3 W after fracture (**d**) (bar: 1 mm). The cartilage is stained purple and bone tissue is stained pink. The percentage of chondrocyte areas within the callus were measured using Image pro plus. (**e**) Data are shows as the mean ± SD. *: p < 0.05. LT-HG positive; n = 10, LT-HG negative; n = 10

The healing process of the fracture site was observed histologically. In the HT-LG^–^ group at 2 weeks after fracture (2 W), a callus was formed around the fracture site, blood vessels had formed, and chondrocyte areas and trabecular bone, including osteoblasts and osteoclasts, had formed. At 3 weeks after fracture (3 W), almost all of the chondrocytes were resorbed, and blood vessel and trabecular bone formation were accelerated (Fig. 2a, b). In the LT-HG^+^ group, a similar callus was observed at 2 W, but the callus was also seen at 3 W, while the chondrocyte areas persisted (Fig. 2c, d). The ratio of chondroid areas to the callus area was 24% at 2 W and 3% at 3 W in the HT-LG^–^ group, while it was 27% at 2 W and 8% at 3 W in the LT-HG^+^ group. In the LT-HG^+^ group, the chondroid area persisted substantially after 3 W, and the callus resorption process was delayed (*P* < 0.05) (Fig. 2e).

**Fig. 3:**
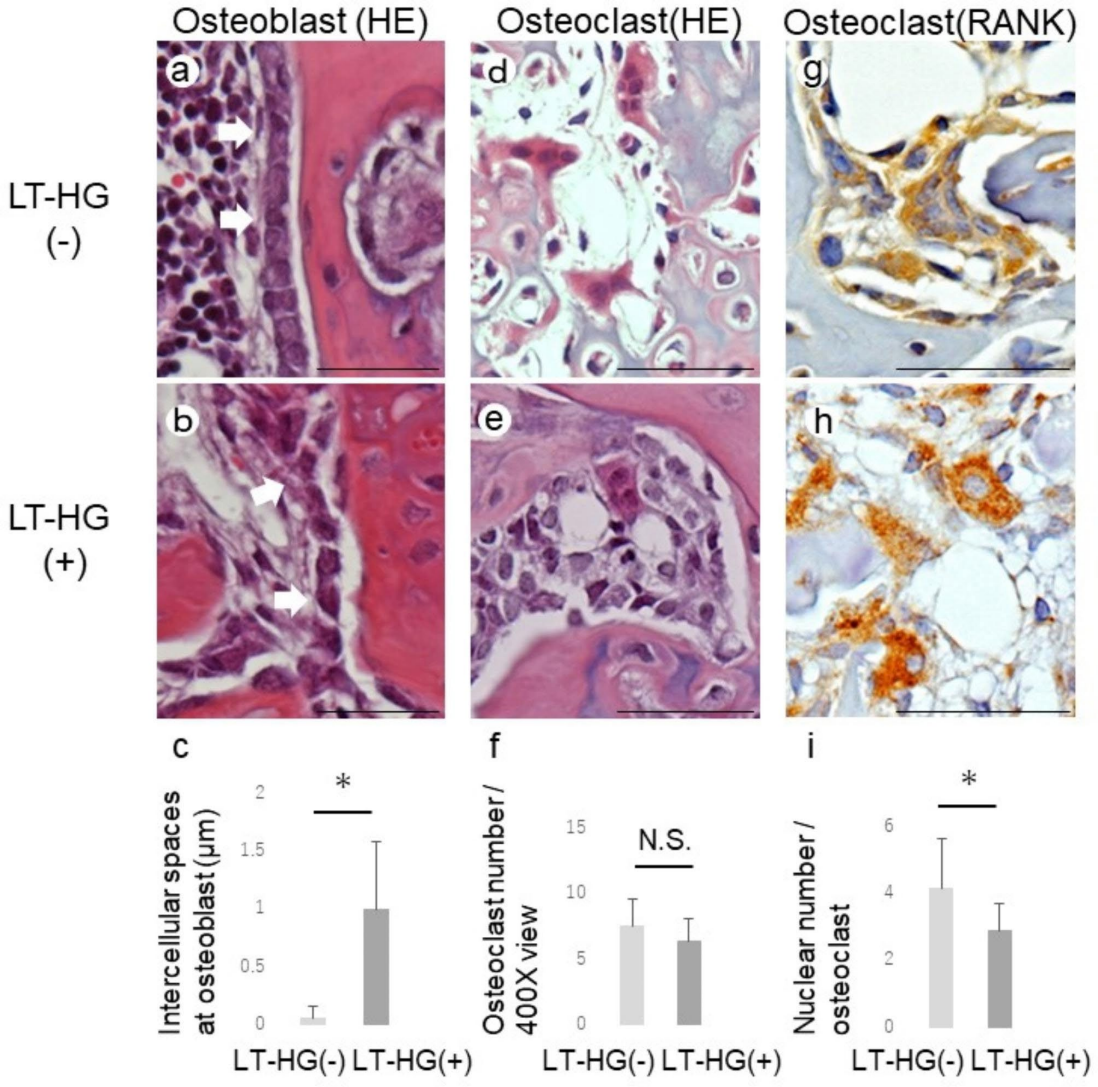
Morphology of osteoblast and osteoclast cells in the callus. Hematoxylin-eosin (HE) images of osteoblasts at 3 weeks after fracture (3 W), without long-term hyperglycaemia (LT-HG) (**a**) and with LT-HG (**b**), White arrows indicate Osteoblasts. Osteoblasts are spindle-shaped cells that arranged on the bone surface. Graph of measured intercellular spaces for osteoblasts at 3 W (**c**); HE images of osteoclasts, without LT-HG (**d**) and with LH-HG (**e**). Graphs of osteoclasts number in the 400X field of view of an optical microscope (**f**); Immunohistochemistry of osteoclasts with anti-RANK antibody, without LT-HG (**g**) and with LT-HG (**h**). Osteoclasts may contain several to dozens of nuclei with eosinophilic cytoplasm. Graph of the number of nuclei per osteoclast. (All bar: 100 μm). *: p < 0.05. N.S: no significant difference. LT-HG positive; n = 10, LT-HG negative; n = 10

In addition, the callus at 2 W was locally observed. In the LT-HG^+^ group, the arrangement of osteoblasts became irregular, the intercellular spaces became significant larger, and the number of cell aggregates tended to decreased (*P* < 0.05) (Fig. 3a, b, c). The average number of osteoclasts was 7.5 in the HT-LG^–^ group and 6.3 in the LT-HG^+^ group under a 400X optical microscope. There was no significant difference in numbers between the two groups (*P* = 0.22) (Fig. 3d, e, f). However, the number of osteoclast nuclei was significantly decreased to 2.9 in the LT-HG^+^ group as compared to 4.2 in the HT-LG^–^ group (*P* < 0.05) (Fig. 3g, h, i).

**Fig. 4:**
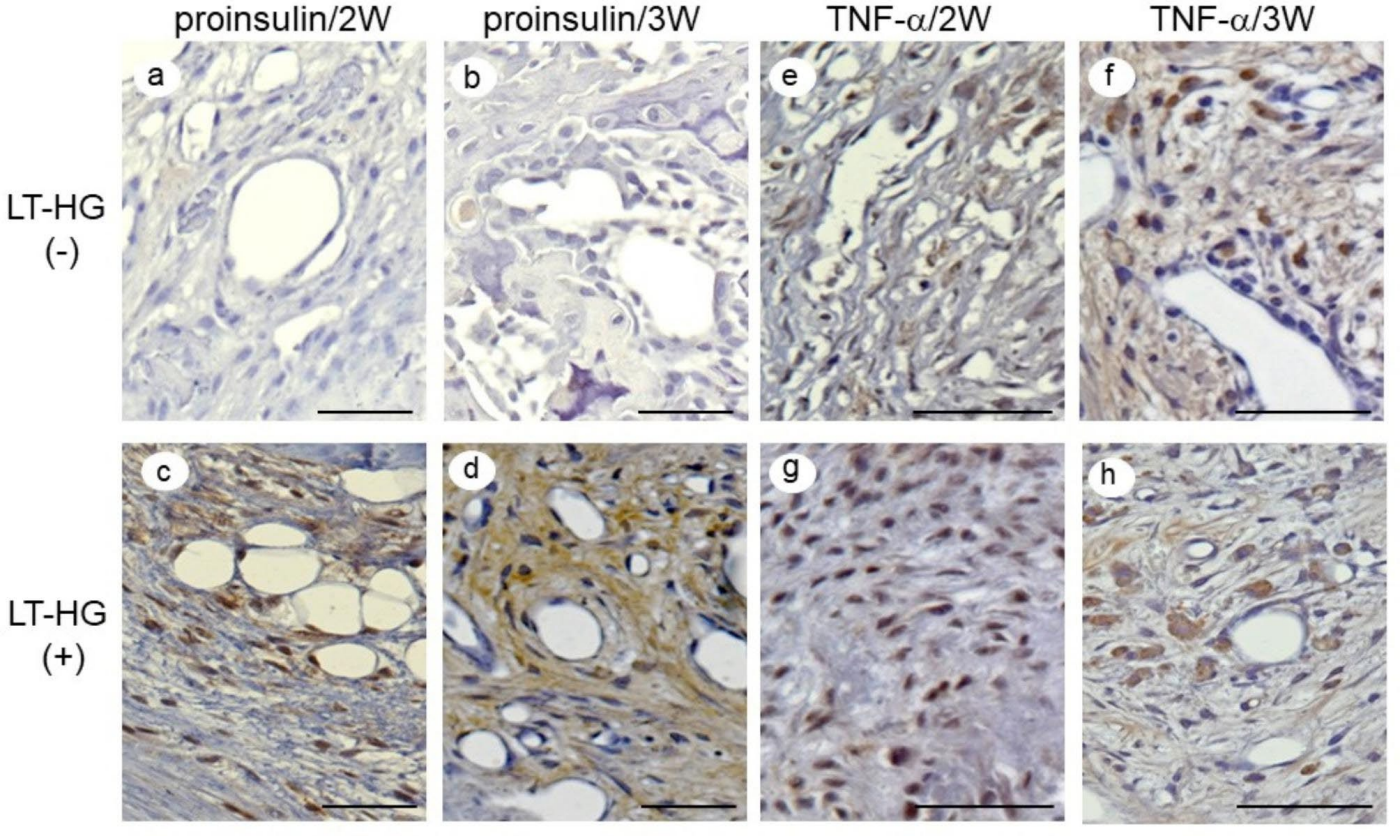
Immunohistochemical staining for proinsulin and TNF-α in the callus. The upper column shows samples without long-term hyperglycaemia (LT-HG) and the lower column shows samples with LT-HG, with staining proinsulin at 2 weeks after fracture (2 W) (**a**, **c**) and 3 weeks after fracture (3 W) (**b**, **d**), TNF-α after 2 W (**e**, **f**) and 3 W (**g**, **h**) (bar: 50 μm). LT-HG positive; n = 10, LT-HG negative; n = 10

Immunostaining revealed that the expression of proinsulin was negative in the HT-LG^–^ group (Fig. 4a, b). In contrast, in the LT-HG^+^ group, the BMDCs surrounding the neovascular nests were strongly positive for proinsulin at 2 and 3 W (Fig. 4c, d). In addition, the hypertrophic chondrocytes adjacent to the trabecular bone and bone tissue were negative for proinsulin expression even in the LT-HG^+^ group. TNF-α expression was weakly positive in the chondrocytes adjacent to bone tissue and strongly positive in BMDCs around small vessels in both groups. In the HT-LG^–^ group, the number of positive cells tended to decrease at 3 W as compared to at 2 W, while in the LT-HG^+^ group, the number of positive cells tended to increase at 2 and 3 W (Fig. 4e, f, g, h; Table 1).

**Table 1:** Summary of Immunohistochemical Staining Results

**proinsulin**				
		localization	2 W	3 W
LT-HG	(-)	Chondrocytes	−	−
		BMDCs	−	−
	(+)	Chondrocytes	−	−
		BMDCs	++	++
**TNF-α**				
		localization	2 W	3 W
LT-HG	(-)	Chondrocytes	±	±
		BMDCs	+	+
	(+)	Chondrocytes	+	+
		BMDCs	++	+

TNF, tumor necrosis factor; LT-HG, long term hyperglycemia; BMDCs, bone marrow derived cells; 2 W, 2weeks after fracture; 3 W, 3weeks after fracture; **++**, strongly positive; **+**, moderately positive; ±, weakly positive; **–**, negative staining, semi-quantitative analysis conducted according to the method described by Song J et al. and Yayama T, et al. [29, 30]

**Fig. 5:**
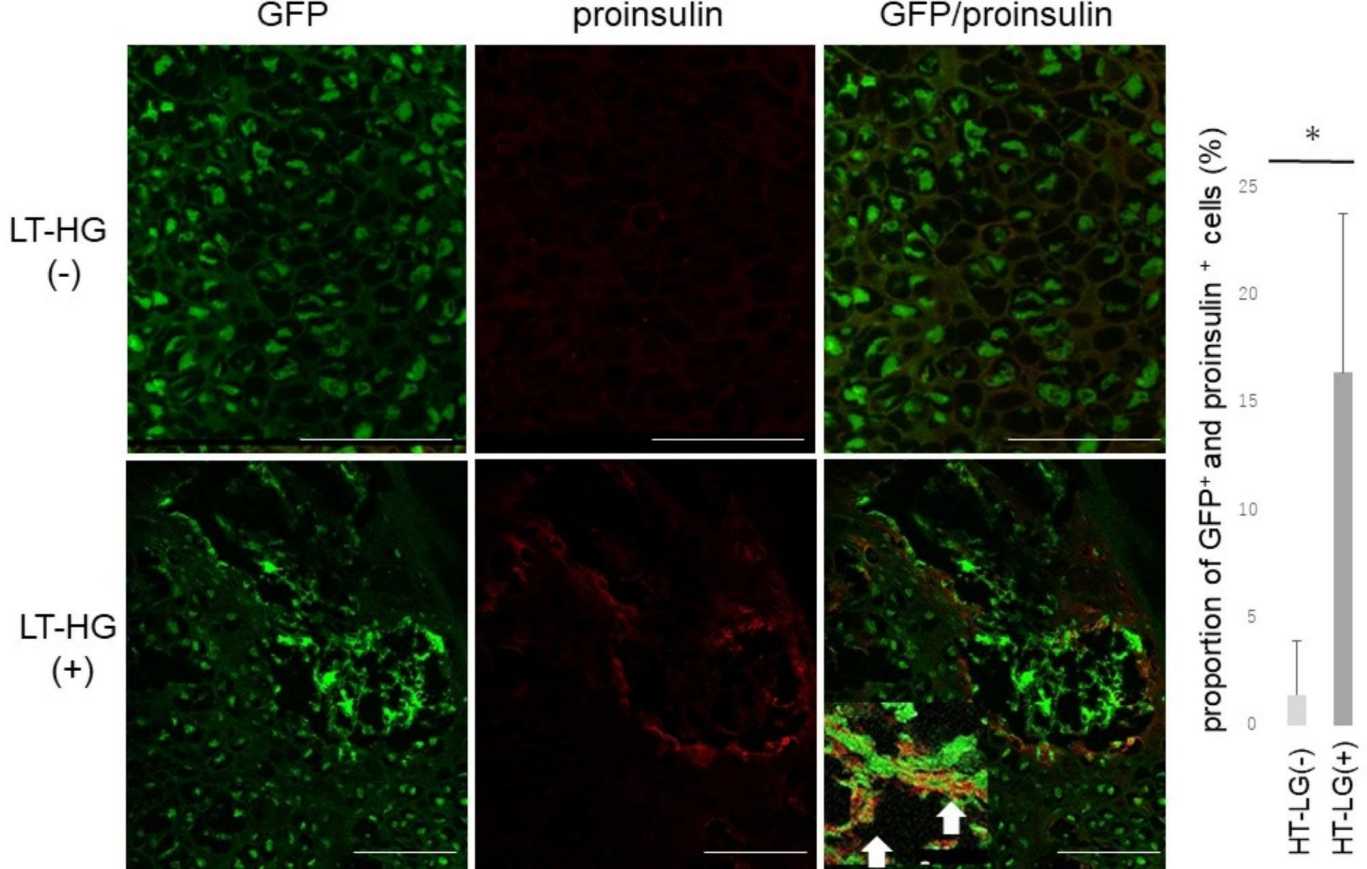
Fluorescent staining of the callus after green fluorescent protein (GFP) bone marrow transplantation. Double image of proinsulin and GFP staining. The upper column shows samples without long-term hyperglycemia (LT-HG) and the lower column shows samples with LT-HG. Proinsulin-positive cells are stained red and bone marrow-derived cells are stained green. The white arrow in the right side panel shows a double image of proinsulin and GFP. (bar: 100 μm). The graph shows the proportion of GFP-positive cells (GFP+) and proinsulin-positive cells. *: p < 0.05. GFP and LT-HG positive; n = 5, GFP positive and LT-HG negative; n = 5

**Fig. 6:**
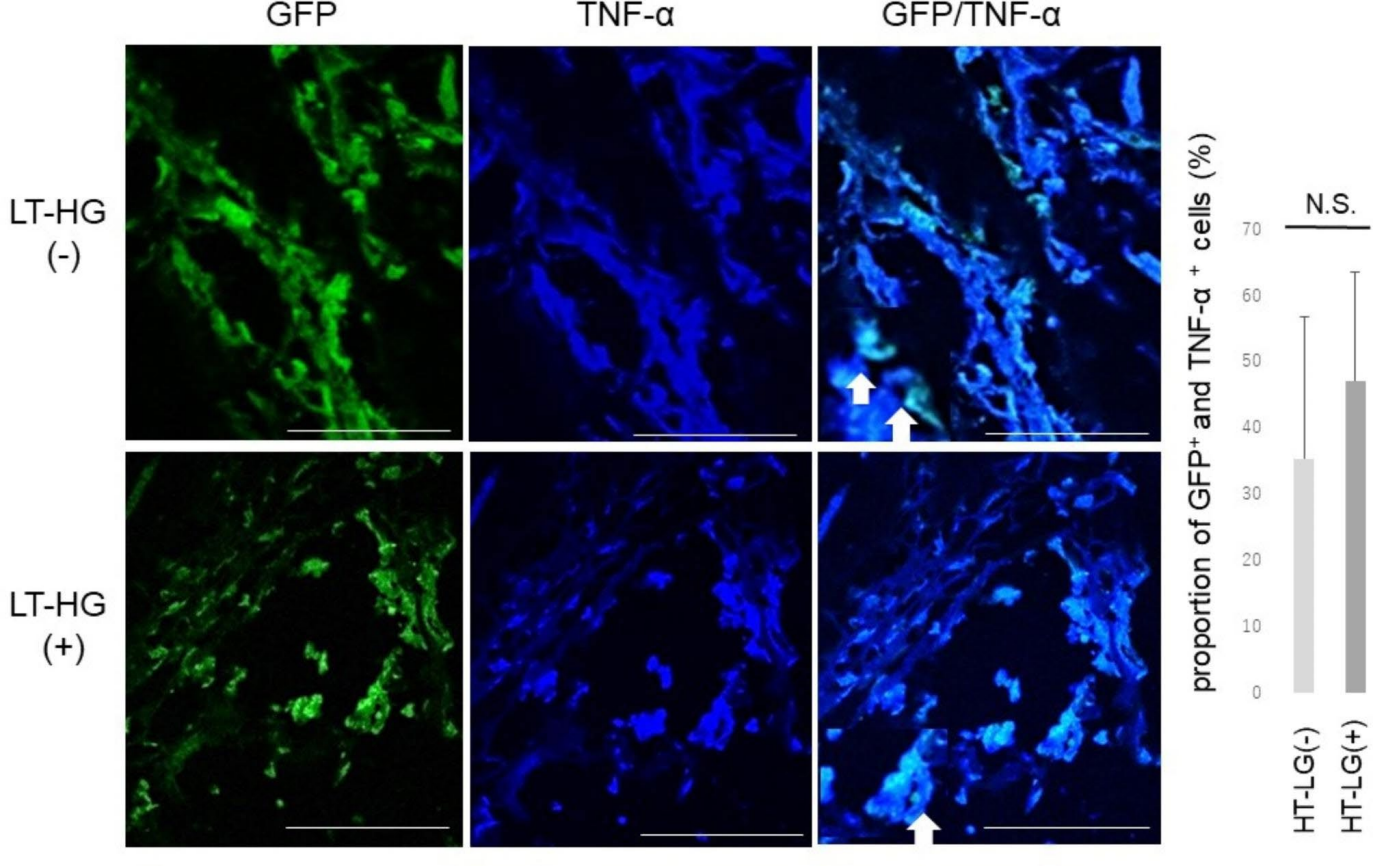
Fluorescent staining of the callus after green fluorescent protein (GFP) bone marrow transplantation. Double image of TNF-α and GFP. The upper column shows samples without long-term hyperglycaemia (LT-HG) and the lower column shows samples with LT-HG. TNF-α-positive cells are stained blue, and bone marrow-derived cells are stained green. The white arrow in the right side panel shows the double image of TNF-α and GFP. (bar: 100 μm). The graph shows the proportion of GFP-positive cells (GFP+) and TNF-α-positive cells. N.S: no significant difference. GFP and LT-HG positive; n = 5, GFP positive and LT-HG negative; n = 5

After BMT of GFP-positive cells, many GFP-positive cells could be observed in calluses in both HT-LG^–^and LT-HG^+^ groups. Overlaying the immunolocalization of GFP-positive cells and proinsulin, GFP-positive and proinsulin-positive cells were identified among the BMDCs population, and the proportion of GFP-positive cells (GFP^+^) and proinsulin-positive cells was 1.4% in the GFP^+^ / HT-LG^–^ and 15% in the GFP^+^ / LT-HG^+^ groups (*P* < 0.05) (Fig. 5). GFP^+^ / TNF-α^+^ cells were also found in BMDCs around the neovascular nests, accounting for 35% of the total GFP^+^ / HT-LG^–^ and 47% GFP^+^ / LT-HG^+^ (*P* = 0.07) (Fig. 6). These results suggest that the proinsulin-positive and TNF-α-positive BMDCs that collect in the calluses of the LT-HG groups are of BM origin.

## Discussion

The primary feature of this study was that a LT-HG model was created and validated. As compared to the study by Kasahara et al. which used a short-term hyperglycaemia model, the LT-HG model presented herein showed significant thinning of the femoral cortex (Supplemental Data. c) and increased BM adiposity and bone fragility against blunt external force [13]. These results better approximate the true clinical conditions of DM patients.

It has been widely reported that bone formation reduces under hyperglycaemic conditions, while recently it has also been reported that the proliferative capacity of human BMCs is reduced after 2 weeks of culture under hyperglycaemia [15, 16], and that induction of differentiation of mouse BMCs under hyperglycaemia promotes apoptosis of BMCs [10]. In addition, some studies have reported that the differentiation of BMCs into adipocytes is promoted by hyperglycaemia and, while differentiation into osteoblasts is impaired. Inhibition of osteoblast maturation in cultured mouse tissue [7, 8], impaired osteoblast differentiation, and reduced mineralization in mouse BMCs have also been reported [10]. However, these reports are from in vitro models in which tissue or cells themselves are cultured under hyperglycaemic conditions, and there are no reports of in vivo studies in which LT-HG fracture models were created and studied.

Regarding bone resorption capacity under hyperglycaemia, previous studies have been reported that there is no difference in osteoclast function between hyperglycaemic and nonhyperglycaemic mice because there are no significant differences in bone metabolism markers (serum TRAP5b, urinary DPD) or proteolytic enzymes (cathepsin K) [7, 8]. Conversely, in the bone calluses of mice treated with STZ injection and hyperglycaemic for 3 weeks, osteoclast function is reportedly enhanced due to an increase in TRACP cells, aggrecanases (ADAMTS), and collagenases (MMP-13,16) [17]. In a human control study, type 2 DM patients were reported to have lower bone resorption capacity due to lower bone metabolism markers (serum TRAP5b, serum CTX) [18]. As described above, there is currently no consensus regarding osteoclast function and bone resorption capacity under hyperglycaemic conditions, meaning that findings from LT-HG models, as presented herein, are considered important. Based on research by Kasahara et al., we believe that osteoclast function is reduced [13].

The callus in the LT-HG model of this study was characterized by delayed chondrocyte resorption, disrupted osteoblast aggregation, impaired multinucleation of osteoclasts, and narrowing of neovascular vessels. This study also found that the arrangement of osteoblasts became irregular, the gap between cells was large, and the number of aggregated cells tended to be small. These results, agree with previous experiments with cultured cells, suggesting that hyperglycaemia exposure impairs BMCs, resulting in impaired differentiation and maturation into osteoblasts and abnormal osteoblast morphology. This study compared results at 2–3 weeks after fracture, as Kasahara et al. found the greatest difference in callus characterization during this time [13]. As a result, we observed for the first time that osteoclasts in the callus did not increase in cell number and had fewer nuclei when LT-HG was maintained. This clearly indicates that BMCs are impaired under LT-HG exposure, and that bone resorption capacity is reduced due to the impaired differentiation and function of osteoclasts and osteoclasts. The results of this study suggest that the bone metabolic capacity of the LT-HG model has decreased function in both bone formation and bone resorption.

Insulin producing cells normally occur only in the pancreas and thymus [19]. However, Kojima et al. reported that hyperglycaemia in mice and rats induces the expression of proinsulin positive cells, an insulin transcript, in the liver, adipose tissue, and bone marrow, and that hyperglycaemia induces the appearance of proinsulin-producing cells in the BM [20]. Terashima et al. reported that diabetic neuropathy in mice is caused by the cell fusion of proinsulin-positive cells in the sciatic nerve and dorsal root ganglia, with the fused cells expressing TNF-α and undergoing a high rate of apoptosis [12]. Diabetic neuropathy in STZ-treated DM mice is also reported to require proinsulin-positive BMCs to produce TNF-α [21]. This result suggests that proinsulin-positive cells appear at various sites. In the present study, the expression of proinsulin and TNF-α was also observed in the callus area in the LT-HG model, suggesting that the BMCs themselves may be damaged by the same mechanism as diabetic neuropathy, resulting in a prolonged fracture healing process. Together, these results suggest that proinsulin may be a potential biomarker for the early diagnosis of diabetic tissue damage.

Regarding the role of TNF-α, some reports from BMT experiments in DM mice indicate that diabetic BMCs themselves may express TNF-α and cause glomerular damage [22], and that diabetic BMCs fuse with hepatocytes, resulting in TNF-α production and causing diabetic hepatocyte injury [23]. Diabetes-enhanced TNF-α also reduces MSC proliferation, increases MSC apoptosis, and inhibits osteoblast differentiation via Indian Hedgehog signaling [24, 25]. Excessive secretion of TNF-α induced by DM could decrease the concentration of MSCs in cortical callus at the initial stage of fracture healing [26]. In the present study, the increase in proinsulin-positive and TNF-α-positive cells in BMDCs in the callus were thought to impair the proliferation of BMCs and their differentiation into osteoblasts, thereby reducing their osteogenic potential.

Induction of mesenchymal cells (MCs) from a local site such as the periosteum or BM is important for fracture healing. In addition, one of the important roles of MCs is their involvement in hematopoietic stem cell (HSC) proliferation and maintenance of HSC progenitors [27, 28]. As the callus at 2 W post-fracture in the present study was composed of many GFP-positive BMDCs, the induction of BMDCs into the matrix with angiogenesis was considered important in the fracture healing process. In addition, there were more proinsulin-positive and TNF-α-positive BMDCs in the LT-HG^+^ group, suggesting that LT-HG exposure may cause abnormalities in the BMDCs themselves.

One limitation of this study is that it did not consider biological responses such as the release of cytokines due to the external factors influencing fracture creation. To minimize the effects of fracture to the greatest extent possible, an osteotomy model was used instead of a blunt fracture model, in addition to soft tissue protection. The present study was limited to the second and third weeks after the fracture. However, in a prior study by Kasahara et al. [13], the greatest difference in callus formation was observed 2–3 weeks after fracture. In addition, Kayal et al. reported a markedly increased expression of RANKL, TRACP, and TNF-α at 12 and 16 days after fracture in DM mice [17]. Therefore, the evaluation period for this study was judged to be sufficient. Future studies, however, may wish to address different time points after fracture.

## Conclusions

Proinsulin and TNF-α positive BMDCs were found to be induced in the LT-HG condition, while bone formation and bone resorption functions were impaired. From the perspective of the enhancement of inflammatory cytokines and impaired glucose metabolism, we believe that the results of this study provide useful information for the development of new diagnostic methods, such as biomarkers for bone metabolism dysfunction caused by diabetes, and for the development of therapeutic methods, such as anti-cytokine therapy.

## Electronic supplementary material

Below is the link to the electronic supplementary material.

Supplementary Material 1

Supplementary Material 2

Supplementary Material 3

Supplementary Material 4

## Data Availability

All data generated or analyzed during this study are included in this published article or can be made available from the corresponding author on reasonable request.

## List of abbreviations

2W: 2 weeks after fracture
3W: 3 weeks after facture
BGL: Blood glucose level
BM: Bone marrow
BMCs: Bone marrow cells
BMDCs: Bone marrow derived cells
BMT: Bone marrow transplantation
BW: Body weight
DM: Diabetes mellitus
GDP: Gross domestic product
GFP: Green fluorescent protein
HE: Hematoxylin-eosin
HSC: Hematopoietic stem cell
HT-GH (-): Non-hyperglycaemia control group
IHC: Immunohistochemistry
LT-HG: Long-term hyperglycaemia
MCs: Mesenchymal cells
MSC: Mesenchymal stem cells
PBS: Phosphate buffer saline
SD: Standard deviation
STZ: Streptozotocin
TNF: Tumor necrosis factor
