# Supplementary material for: Expression of proinflammatory cytokines and proinsulin by bone marrow-derived cells for fracture healing in long-term diabetic mice
Source: BMC Musculoskelet Disord. 2023 Jul 18;24:585. doi: 10.1186/s12891-023-06710-5 (PMC10355075; doi:10.1186/s12891-023-06710-5)
Supplement: Supplementary file 1 — Supplementary Material 1 [file 12891_2023_6710_MOESM1_ESM.pptx]

## Slide 1
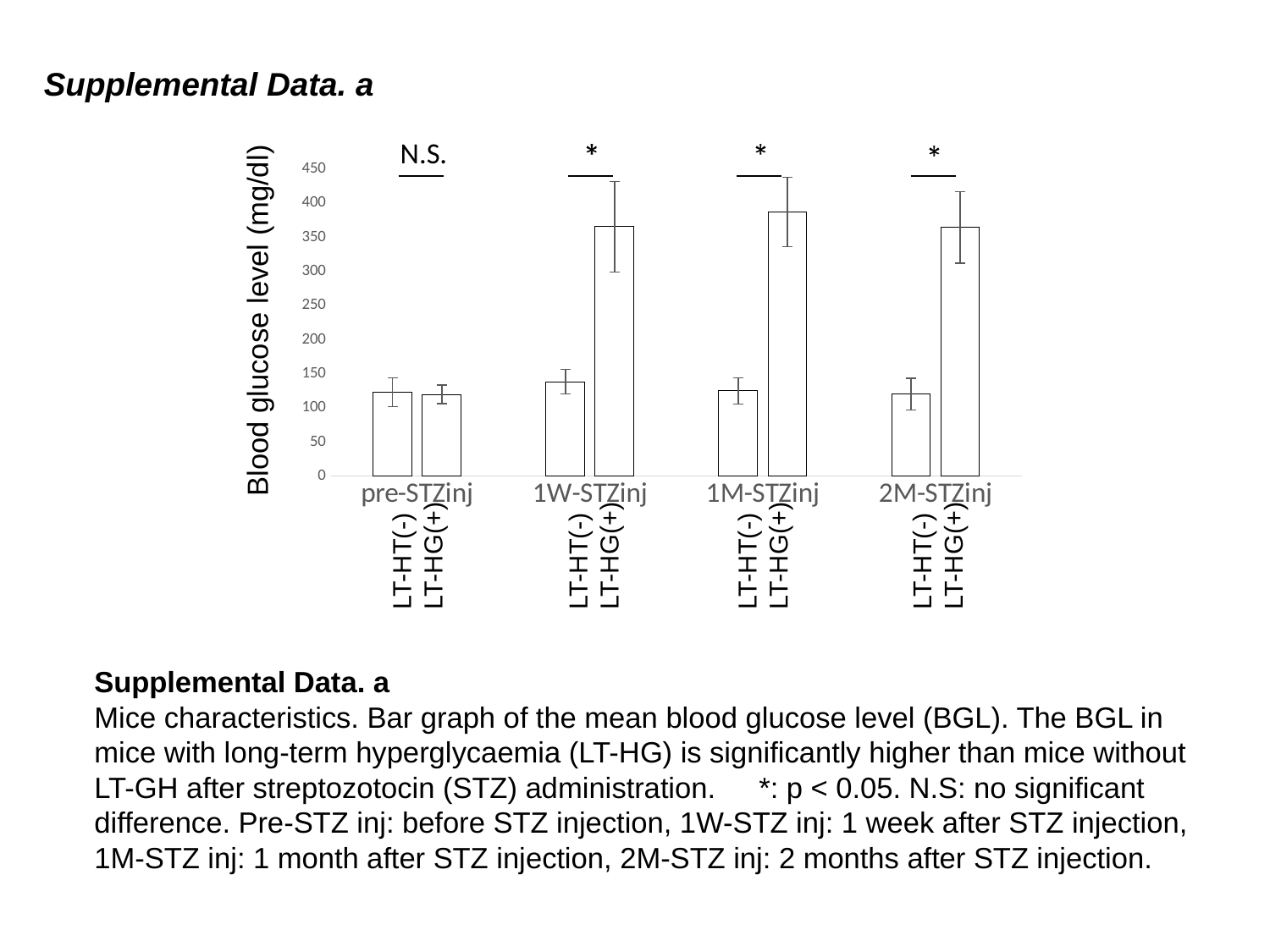

Supplemental Data. a
N.S.
### Chart
| Category | LT-HG(ｰ) | LT-HG(+) |
|---|---|---|
| pre-STZinj | 122.8 | 119.6 |
| 1W-STZinj | 138.3 | 365.4 |
| 1M-STZinj | 125.0 | 387.1 |
| 2M-STZinj | 120.0 | 364.4 |Blood glucose level (mg/dl)
LT-HT(-)
LT-HG(+)
LT-HT(-)
LT-HG(+)
LT-HT(-)
LT-HG(+)
LT-HT(-)
LT-HG(+)
Supplemental Data. a
Mice characteristics. Bar graph of the mean blood glucose level (BGL). The BGL in mice with long-term hyperglycaemia (LT-HG) is significantly higher than mice without LT-GH after streptozotocin (STZ) administration.　*: p < 0.05. N.S: no significant difference. Pre-STZ inj: before STZ injection, 1W-STZ inj: 1 week after STZ injection, 1M-STZ inj: 1 month after STZ injection, 2M-STZ inj: 2 months after STZ injection.
