# Supplementary material for: Expression of proinflammatory cytokines and proinsulin by bone marrow-derived cells for fracture healing in long-term diabetic mice
Source: BMC Musculoskelet Disord. 2023 Jul 18;24:585. doi: 10.1186/s12891-023-06710-5 (PMC10355075; doi:10.1186/s12891-023-06710-5)
Supplement: Supplementary file 2 — Supplementary Material 2 [file 12891_2023_6710_MOESM2_ESM.pptx]

## Slide 1
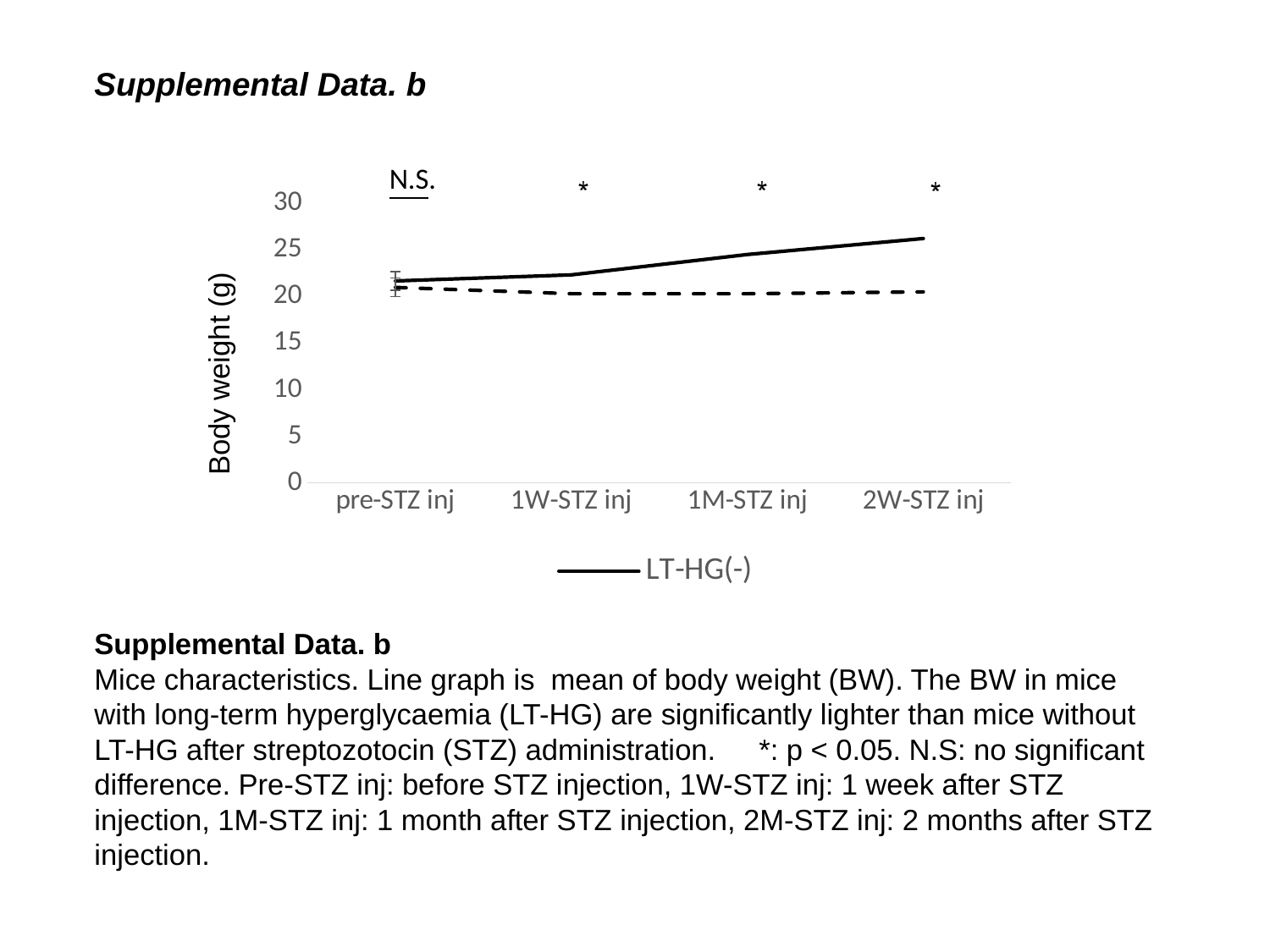

Supplemental Data. b
N.S.
### Chart
| Category | LT-HG(-) | LT-HG(+) |
|---|---|---|
| pre-STZ inj | 21.667058823529405 | 20.976666666666667 |
| 1W-STZ inj | 22.321875000000002 | 20.296666666666667 |
| 1M-STZ inj | 24.504545454545454 | 20.296666666666667 |
| 2W-STZ inj | 26.220555555555556 | 20.501176470588234 |Body weight (g)
Supplemental Data. b
Mice characteristics. Line graph is mean of body weight (BW). The BW in mice with long-term hyperglycaemia (LT-HG) are significantly lighter than mice without LT-HG after streptozotocin (STZ) administration.　*: p < 0.05. N.S: no significant difference. Pre-STZ inj: before STZ injection, 1W-STZ inj: 1 week after STZ injection, 1M-STZ inj: 1 month after STZ injection, 2M-STZ inj: 2 months after STZ injection.
