# Supplementary material for: Expression of proinflammatory cytokines and proinsulin by bone marrow-derived cells for fracture healing in long-term diabetic mice
Source: BMC Musculoskelet Disord. 2023 Jul 18;24:585. doi: 10.1186/s12891-023-06710-5 (PMC10355075; doi:10.1186/s12891-023-06710-5)
Supplement: Supplementary file 3 — Supplementary Material 3 [file 12891_2023_6710_MOESM3_ESM.pptx]

## Slide 1
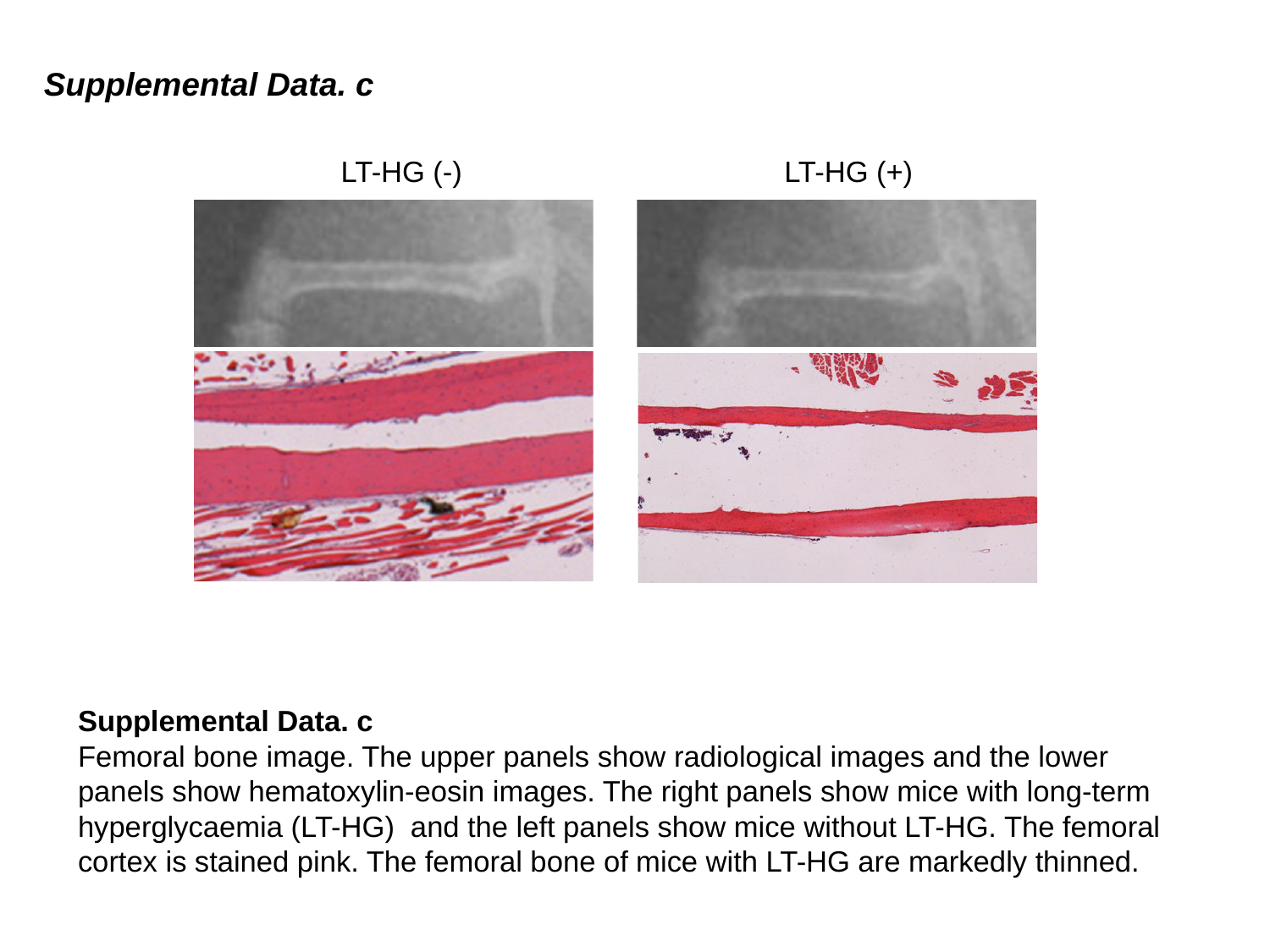

Supplemental Data. c
LT-HG (-)
LT-HG (+)
Supplemental Data. c
Femoral bone image. The upper panels show radiological images and the lower panels show hematoxylin-eosin images. The right panels show mice with long-term hyperglycaemia (LT-HG) and the left panels show mice without LT-HG. The femoral cortex is stained pink. The femoral bone of mice with LT-HG are markedly thinned.
