# Supplementary material for: Expression of proinflammatory cytokines and proinsulin by bone marrow-derived cells for fracture healing in long-term diabetic mice
Source: BMC Musculoskelet Disord. 2023 Jul 18;24:585. doi: 10.1186/s12891-023-06710-5 (PMC10355075; doi:10.1186/s12891-023-06710-5)
Supplement: Supplementary file 4 — Supplementary Material 4 [file 12891_2023_6710_MOESM4_ESM.pptx]

## Slide 1
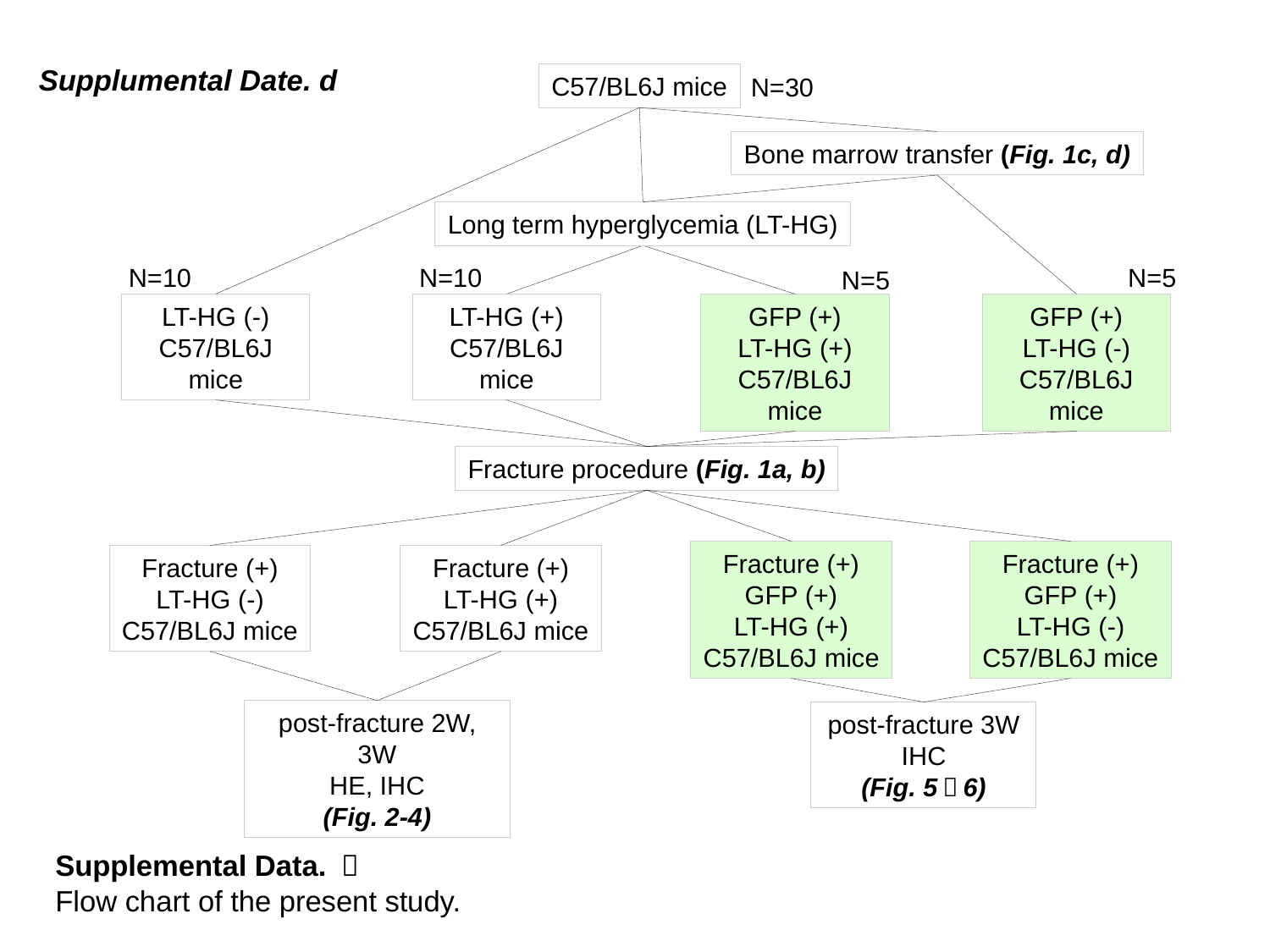

Supplumental Date. d
C57/BL6J mice
N=30
Bone marrow transfer (Fig. 1c, d)
Long term hyperglycemia (LT-HG)
N=10
N=10
N=5
N=5
LT-HG (-)
C57/BL6J mice
LT-HG (+)
C57/BL6J mice
GFP (+)
LT-HG (+)
C57/BL6J mice
GFP (+)
LT-HG (-)
C57/BL6J mice
Fracture procedure (Fig. 1a, b)
Fracture (+)
GFP (+)
LT-HG (+)
C57/BL6J mice
Fracture (+)
GFP (+)
LT-HG (-)
C57/BL6J mice
Fracture (+)
LT-HG (-)
C57/BL6J mice
Fracture (+)
LT-HG (+)
C57/BL6J mice
post-fracture 2W, 3W
HE, IHC
(Fig. 2-4)
post-fracture 3W
IHC
(Fig. 5，6)
Supplemental Data. ｄ
Flow chart of the present study.
